# Construction of Chimeric Dual-Chain Avidin by Tandem Fusion of the Related Avidins

**DOI:** 10.1371/journal.pone.0020535

**Published:** 2011-05-31

**Authors:** Tiina A. Riihimäki, Sampo Kukkurainen, Suvi Varjonen, Jarno Hörhä, Thomas K. M. Nyholm, Markku S. Kulomaa, Vesa P. Hytönen

**Affiliations:** 1 Institute of Biomedical Technology, University of Tampere and Tampere University Hospital, Tampere, Finland; 2 Department of Biological and Environmental Science, University of Jyväskylä, Jyväskylä, Finland; 3 Department of Biochemistry and Pharmacy, Åbo Akademi University, Turku, Finland; Leeds Institute of Molecular Medicine, United Kingdom

## Abstract

**Background:**

Avidin is a chicken egg-white protein with high affinity to vitamin H, also known as D-biotin. Many applications in life science research are based on this strong interaction. Avidin is a homotetrameric protein, which promotes its modification to symmetrical entities. Dual-chain avidin, a genetically engineered avidin form, has two circularly permuted chicken avidin monomers that are tandem-fused into one polypeptide chain. This form of avidin enables independent modification of the two domains, including the two biotin-binding pockets; however, decreased yields in protein production, compared to wt avidin, and complicated genetic manipulation of two highly similar DNA sequences in the tandem gene have limited the use of dual-chain avidin in biotechnological applications.

**Principal Findings:**

To overcome challenges associated with the original dual-chain avidin, we developed chimeric dual-chain avidin, which is a tandem fusion of avidin and avidin-related protein 4 (AVR4), another member of the chicken avidin gene family. We observed an increase in protein production and better thermal stability, compared with the original dual-chain avidin. Additionally, PCR amplification of the hybrid gene was more efficient, thus enabling more convenient and straightforward modification of the dual-chain avidin. When studied closer, the generated chimeric dual-chain avidin showed biphasic biotin dissociation.

**Significance:**

The improved dual-chain avidin introduced here increases its potential for future applications. This molecule offers a valuable base for developing bi-functional avidin tools for bioseparation, carrier proteins, and nanoscale adapters. Additionally, this strategy could be helpful when generating hetero-oligomers from other oligomeric proteins with high structural similarity.

## Introduction

Improving the performance and accuracy of molecular tools used in life science research is essential for developing better and more precise methods. Chicken avidin (AVD) and its bacterial analogue streptavidin (SA) from *Streptomyces avidinii*, collectively called (strept)avidin, are proteins widely used in life science research applications. (Strept)avidin has been used for quantitative measurements by radioligand-binding methods [1], enzyme assays [2], [3] and photometric/fluorometric methods [4]–[6]. AVD has also been successfully used in biosensors as an immobilization platform [7]. The specific characteristics of AVD, such as the high positive charge (pI 10.5) and high biotin-binding affinity (K_d_≈10^−15^ M), have resulted in a number of different drug-targeting applications that use the (strept)avidin-biotin interaction [8].

Although widely used, (strept)avidin's homotetrameric structure restricts its use in some applications. To overcome this limitation, dual-chain avidin (dcAVD) was generated [9] by fusing two circularly permuted chicken avidin monomers into one polypeptide chain. This molecular engineering approach produced a protein that resembles wt avidin in 3-D structure [10] but allows independent manipulation of ligand-binding sites within a single protein particle. To create dual-affinity derivatives, dcAVD was modified by site-directed mutagenesis. This form of dcAVD exhibited a tight biotin affinity for two binding sites, whereas the other two binding sites had reduced affinity due to the mutations [11]. Recently, a point mutation S16C was targeted into one of the biotin-binding sites of dcAVD [12]. The introduction of a chemically active thiol group into the binding site made it possible to selectively control the biotin-binding activity of the dcAVD domains by a mild chemical treatment.

Despite recent improvements, some challenges that could limit the use of dcAVD persist. For instance, PCR amplification of the dcAVD-encoding sequence has been challenging because primers recognize complementary sequences from both subunits, which would produce several different PCR products. This same issue also limits targeted mutagenesis of dcAVD. This study uses a new approach for developing the dcAVDs. Instead of constructing a tandem gene by combining two differently circularly permuted chicken avidins [9] or streptavidins [13], two related avidin genes were used as building blocks for the chimeric tandem fusion. The raw materials of the chimeras were selected from a group of homologous proteins. Streptavidin (SA), the most widely used protein in biotechnological applications in the avidin family, and avidin-related proteins 2 and 4 (AVR2 and AVR4) [14], [15] were selected as fusion partners for the circularly permuted chicken avidin. By combining homologous genes into the chimeric tandem gene, we were able to address the problems associated with the original dcAVD. According to our knowledge, this is the first study showing such forced hybridization of different types of biotin-binding proteins into an oligomeric assembly.

## Results and Discussion

**Figure 1:**
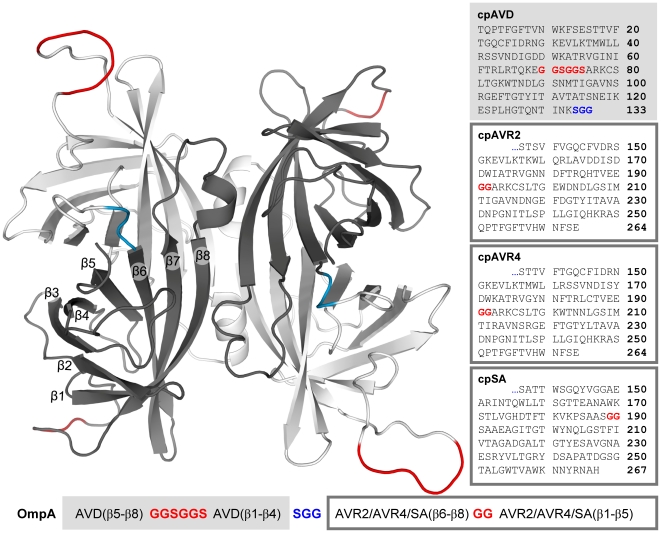
The homology model of dcAVD/AVR4 and the sequences of chimeric dcAVDs. The molecular model of dcAVD/AVR4 is generated by exploiting the existing 3-D structures of the dcAVD and AVR4. In the model, cpAVD is illustrated in light gray, and cpAVR4 is illustrated in dark gray. Amino acid sequence of cpAVD is in the light gray box, and the amino acid sequences of cpAVR2, cpAVR4, and cpSA are in the dark gray boxes. The linkers and the corresponding linker sequences connecting the original termini are shown in red. The linkers connecting the circularly permuted subunits and the corresponding linker sequences are shown in blue. The schematic representation of the protein expression cassette is in the bottom of the figure.

The chimeric dcAVD fusions were designed according to previously described principles [9]. The loop connecting the original termini of the circularly permuted SA, AVR4, and AVR2 was shortened when compared to the original dcAVD, as presented in Figure 1. This shortening was performed because the loop was largely invisible in the X-ray analysis of dcAVD, indicating high mobility of the loop region [10].

### Chimeric dcAVD genes performed well in PCR

Two conditions were used to evaluate performance of the dcAVD forms in PCR amplification. When primers recognizing the sequence that flank the tandem fusion gene were used, the amplification resulted in no notable differences between dcAVD and chimeric fusions. Amplification of the four dual-chain genes produced appropriately sized (∼1000 bp) PCR products; however, a shorter product (700 bp) was clearly observed, except for dcAVD/SA (not shown). The short product could be a result of the amplification of a self-hybridized tandem gene. No clear differences were observed for reactions using the different thermal cycling parameters.

**Figure 2:**
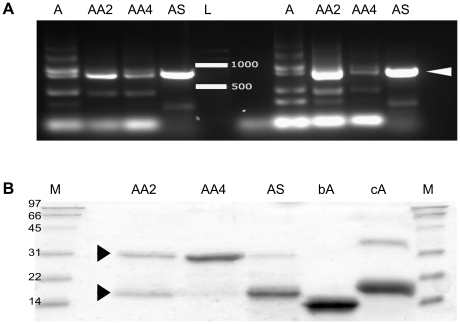
The performance of the chimeric dual-chain avidins in the PCR analysis and in *E. coli* expression. The usability of the generated chimeric dual chain avidin fusions was evaluated by amplifying the fusion genes by PCR and by expressing the proteins in *E. coli*. A) In the PCR analysis with primers recognizing regions inside the chimeric dcAVD genes (PCR 2), a clear difference between the behavior of the dcAVD genes (A) and the chimeric fusion genes (AA2, dcAVD/AVR2; AA4, dcAVD/AVR4; SA, dcAVD/SA) was detected. The chimeric fusion genes showed only two main PCR products; the appropriately sized product had the highest concentration. When the dcAVD gene was used as a template, several different-sized products were produced. The results from the PCR2 reaction (see Table S1) from two different conditions (I, II) are shown in the figure. (L, 1 kb DNA ladder). B) SDS-PAGE analysis of the purified chimeric dcAVDs showed that dcAVD/AVR4 was the most successfully expressed in its functional form in *E. coli*. The upper arrowhead indicates the location of the intact protein, and the lower arrowhead indicates the location of the proteolytically cleaved product. (M, molecular weight standard; AA2, dc-AVD-AVR2; AA4, dc-AVD-AVR4; AS, dc-AVD-SA, bA, chicken avidin control sample (protein expressed in *E. coli* [19]); cA, chicken avidin control sample).

In contrast, when primers recognizing regions inside the tandem fusion gene were used, a clear difference in the behavior between dcAVD and the chimeric fusions was detected (Figure 2A). A number of side products were generated during the PCR amplification of the dcAVD gene, which was probably due to primers binding to multiple positions in the tandem gene and homologous recombination during the amplification process. In contrast, for the chimeric fusions, only two main PCR products were detected, which were the appropriately sized products. The dcAVD/SA showed the most efficient amplification. Using the chimeric fusion genes significantly improves PCR amplification and would allow targeting of mutagenesis to only a part of the gene, such as using the Quik-Change (Stratagene, La Jolla, CA, US) protocol. Moreover, this would enable the broader modification of dcAVD molecules, including targeted random mutagenesis of several amino acids.

### The chimeric fusion dcAVD/AVR4 can be efficiently expressed in *E. coli*

All proteins selected as chimeric fusion partners resemble avidin in their fold and 3-D structure [16]–[18]. Therefore, we assumed that chimeric dual-chain avidins could be produced where one circularly permuted subunit would be based on the avidin sequence, and the other subunit would be based on another biotin-binding protein (AVR2, AVR4, SA). The chimeric dual-chain fusions were produced in *E. coli* using the periplasmic signal peptide from the *Bordetella avium* ompA protein [19]. The chimeric fusion dcAVD/AVR4 produced the best levels of the proteins analyzed, and the protein was almost entirely intact (∼35 kDa) after 2-iminobiotin affinity chromatography (Figure 2B). To further study the usability of this chimeric protein, a pilot-scale expression of the dcAVD/AVR4 protein was performed in a 7.5 L fermentor. The pilot-scale fed-batch fermentation in standard LB medium yielded greater than 5 mg of pure dcAVD/AVR4 protein per liter of production medium with low amounts of protein fragments (Figure S1).

When dcAVD/AVR2 and dcAVD/SA proteins were produced, there was a significant amount of fragmented (∼15 kDa) products in the SDS-PAGE analysis (Figure 2B). The low-sequence identity of SA with avidin (only ∼30%, AVR's identity with avidin ∼80% [20]) may explain the modest performance during the production of the chimeric dual-chain fusion with SA. Because some full-length protein was detected in the SDS-PAGE analysis of dcAVD/SA (Figure 2B), it might be possible to genetically tune this AVD/SA-hybrid to enhance its performance. The possible targets for such optimizations are discussed later in the text.

Size-exclusion chromatography was used to determine the oligomeric state of the produced proteins. The analysis revealed that the dcAVD/AVR4 protein was mostly in a pseudo-tetrameric form (Figure S2). Some higher molecular weight species were also detected, which could be explained by the oligomerization of pseudo-tetramers often detected in wt avidin samples [19]. Size-exclusion chromatography analysis of dcAVD/AVR2 revealed a significant proportion of clearly higher molecular weight species than dcAVD/AVR4. Interestingly, both dcAVD/SA and dcAVD/AVR2 appeared mostly in a pseudo-tetrameric form in gel-filtration analysis (results not shown). This result may indicate that the truncated protein forms observed in the SDS-PAGE analysis (Figure 2B) might be able to form oligomeric species, resulting in homotetrameric proteins. In any case, further studies are needed to better understand the properties of dcAVD/AVR2 and dcAVD/SA. In this study, dcAVD/AVR4 protein was selected to further biochemical analyses.

### Differences in the subunit interfaces reveal possible reasons for the characteristics of chimeric dual-chain avidins

**Figure 3:**
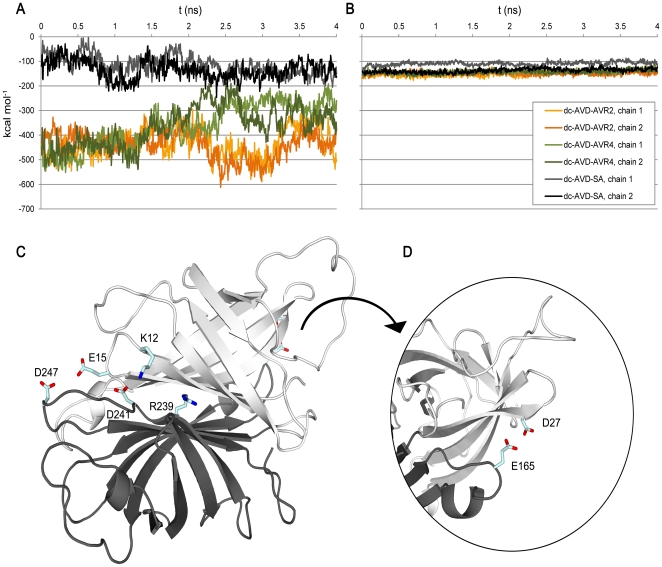
Interactions between the subunit interfaces of chimeric dcAVD fusions by MD simulation. The electrostatic interaction energy was measured between the circularly permuted subunits in chimeric dcAVDs (A). The analysis was performed for both subunit pairs independently, and the interaction energy is plotted over the 4-ns MD simulation. The van der Waals energy between subunits was measured during the simulation time (B). The potential sources of electrostatic repulsion for dcAVD/SA were examined by visual inspection of the MD simulation data. Three clusters of residues (D247-E15-D241, K12-R239 and D27-E165) potentially causing electrostatic repulsion between cpAVD and cpSA were detected. These residues are shown in a liquorice representation (C, D). The figures were prepared using the program PyMOL (www.pymol.org) and numbered according to Figure 1. All the calculations were performed with 5-ps resolution.

To analyze the possible reasons for the behavioral differences of the chimeric dual-chain avidins, the fusion proteins were modeled based on previously determined 3-D structures of dcAVD (PDB 2C4I), SA (PDB 1MK5), and AVRs (AVR2 (PDB 1WBI), AVR4 (PDB 1Y53)). The model of dcAVD/AVR4 is presented in Figure 1. Molecular dynamics (MD) were performed for the predicted chimeric dual-chain avidin models. The simulations were performed in explicit water using the CHARMM force field. The interaction energy during the MD simulation was measured between subunits, which is the most obvious region in the structure that would cause problems in the dcAVD assembly. In the MD simulation analyses, dcAVD/SA had clearly the least favorable electrostatic interaction energy (Figure 3A), whereas there were no significant differences in the van der Waals energies between chimeric dcAVD forms (Figure 3B). A closer inspection of the MD simulation data of dcAVD/SA revealed three putative electrostatically repulsive interactions (Figures 3C and 3D). These residues (D247-E15-D241, K12-R239 and D27-E165) are potential targets for further engineering of dcAVD/SA to improve its characteristics.

**Figure 4:**
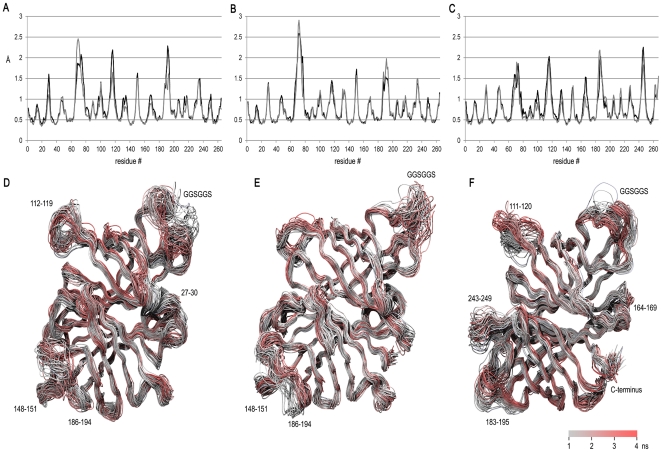
Local dynamics in the chimeric dcAVD fusions measured by MD simulation. To probe the local structural dynamics, the root mean square fluctuation (RMSF) per residue was measured for a short time window (10 ps) for the last 3 ns of the 4-ns MD simulation. The resulting values were averaged and plotted in graphs A–C (A: dcAVD/AVR2; B: dcAVD/AVR4; C: dcAVD/SA). The dynamics of the structure are illustrated by plotting 50 superimposed structural snapshots along the 4-ns simulation (D–F). The loops showing a high amount of structural fluctuation (RMSF>1.5 Å) are indicated by numbers referring to the amino acid sequence (see also Figure 1). The structural snapshots are colored according to timestep, as illustrated by the scale bar (please note that the color scale is illustrative only because of the rendering method). Figures D–F were prepared using the program VMD 1.8.7 [35].

To investigate how the chimeric dcAVD forms differ in terms of loop dynamics in MD simulations, we analyzed a root mean square fluctuation (RMSF), measuring main chain motion for a 10 ps time window (Figure 4). The analysis revealed that the linkers connecting the original termini of the domains (Figure 1, red linkers) were highly mobile, particularly in the avidin domain (GGSGGS, residues 70–75 connecting the original termini). We also detected regions behaving differently between the chimeric dcAVD forms; for dcAVD/SA, there were unique mobile regions in the loop between the strands β7 and β8 (residues 164–169) and in the loop between the strands β4 and β5 (residues 243–249) that corresponded to the potential electrostatic repulsion (Figure 3C and 3D). Overall, however, no dramatic differences were observed in the loop mobility between different dcAVD forms, suggesting that there were no significant problems in the molecular design of the chimeric dcAVDs.

### The chimeric dcAVD/AVR4 showed an increase in thermal stability when compared to dcAVD

A thermal stability assessment of the dcAVD/AVR4 by SDS-PAGE revealed a significant improvement when compared to that of dcAVD. The determined transition temperature of subunit dissociation (T_r_) was 25°C higher for dcAVD/AVR4 (Table 1). The presence of D-biotin in the binding site stabilized the dcAVD/AVR4 quaternary structure, and the determined transition temperature of oligomeric disassembly (T_r_) increased from 65°C to 85°C; however, when compared to dcAVD, the increase in transition temperature due to ligand binding was less (Table 1).

**Table 1:** Transition temperatures of the subunit dissociation (T_r_) and thermal unfolding (T_m_) determined by SDS-PAGE and DSC.

	SDS-PAGE		DSC	
	T_r_ (°C)	T_r_^(+BTN)^ (°C)	T_m_ (°C)	T_m_^(+BTN)^ (°C)
AVD	60^a^	95^a^	84^b^	117^b^
AVR4(C112S)	90	95	110^b^	127^b^
dcAVD	40	75^c^	80^a^	116^a^
dcAVD/AVR4	70	85	*86*/91^d^	*108*/112^d^

a Value from [19];

b [25];

c [9];

d (two-peak analysis was applied to the data, and the minor peak is shown in *italics*).

**Figure 5:**
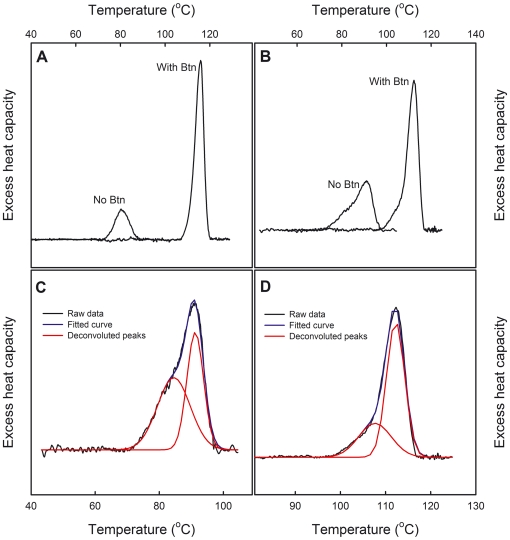
DSC analysis shows the biphasic thermal denaturation mode of the dcAvd/AVR4 protein. Heat-induced unfolding of the proteins was analysed by differential scanning calorimetry. The measured thermogram of dcAVD (A) and dcAVD/AVR4 (B) is shown without and with biotin (With Btn). Deconvoluted thermograms of dcAVD/AVR4 without (C) and with (D) biotin are also shown. The thermogram of dcAVD/AVR4 shows a melting point (T_m_) at 91.3°C (C), which is about 11°C greater than for dcAVD (80.2°C, [11]). The smaller secondary peak shows a melting point at 86.3°C. Biphasic thermal denaturation mode is also detected in the presence of biotin (D); the melting point of the main peak is at 112.3°C, and the secondary peak is at 107.8°C. Interestingly, in the presence of biotin, dcAVD/AVR4 was denatured at a slightly lower temperature compared with dcAVD 115.9°C [11].

Differential scanning calorimetry (DSC) was used to analyse the thermodynamics of the heat-induced unfolding. In a DSC analysis of dcAVD/AVR4, a two-phase melting profile was observed both with and without biotin (Figure 5B). This melting profile was not detected in the dcAVD samples, in which the heat induced unfolding resulted in a single peak in the thermogram (Figure 5A). The thermograms recorded with dcAVD/AVR4 samples were deconvoluted in order to separate the two peaks (Figure 5C & Figure 5D). The main peak in the dcAVD/AVR4 thermogram revealed a melting point (T_m_) at 91.3°C (Figure 5C), which is about 11°C higher compared to that measured for dcAVD (80.2°C). The smaller secondary peak showed a melting point at 86.3°C. In the presence of biotin, the main peak showed a melting point at 112.3°C, and a secondary peak at 107.8°C (Figure 5D). Interestingly, in the presence of biotin, dcAVD/AVR4 was denatured at a lower temperature compared to the melting temperature (115.9°C) of dcAVD. Overall, dcAVD/AVR4 had improved thermal stability in the absence of biotin, but the addition of biotin did not produce as significant thermal stabilization as in the case of dcAVD or wt AVD. Therefore, the exchange of the cp65-subunit of dcAVD with a circularly permuted AVR4 subunit increased the thermal stability of the chimeric fusion protein, while the other domain of the tandem gene remained unchanged (AVD-derived cp54). The lower biotin-binding affinity of the introduced AVR4-derived subunit reflected the thermal stability of the whole fusion protein in the presence of biotin. These results are clear indications of structural cooperativity between the subunits of the tetramer during thermal unfolding; however, previous studies have shown that (strept)avidin has relatively little or no structural cooperativity between subunits [21]–[23]. One reason for the apparently low cooperativity has been attributed to the subunit exchange between partially unfolded proteins [24]. For dcAVD, the covalent attachment of the subunits might block or at least significantly reduce the subunit exchange in the thermal unfolding process. Therefore, the dual chain concept allows a novel type of approach in studies elucidating the unfolding mechanisms of avidin proteins. The unfolding process was irreversible, which is typical for avidin proteins.

### The biotin-binding properties of dcAVD/AVR4 differed from those of the parental proteins

**Figure 6:**
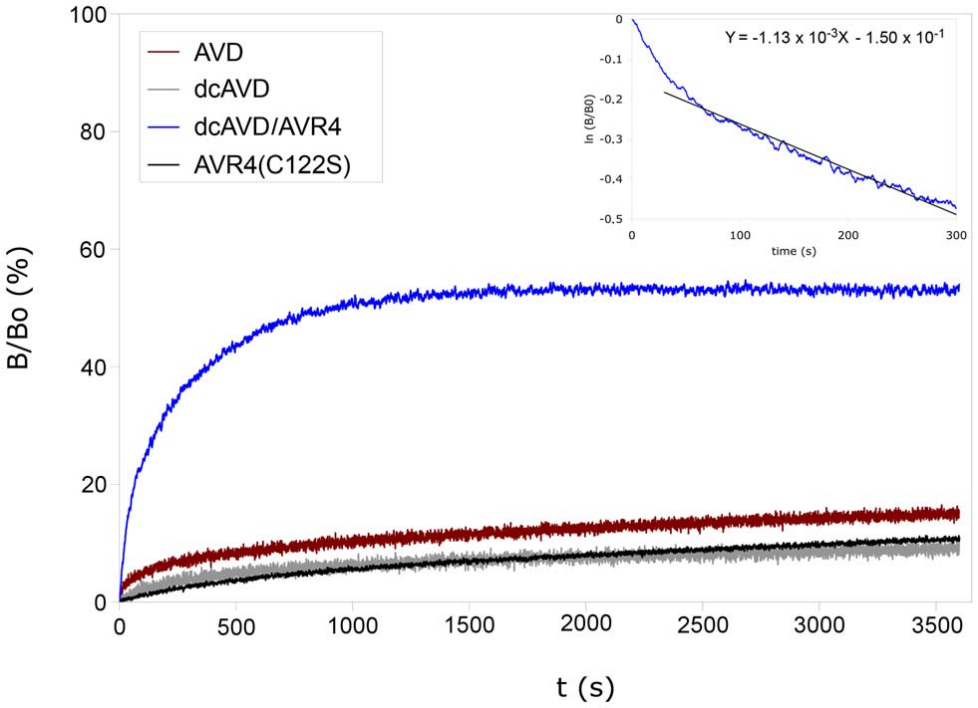
Determination of ligand dissociation kinetics with a fluorescent-biotin conjugate. The dissociation of fluorescently labeled biotin from various avidin forms was studied by replacing the labeled biotin with an excess of free biotin. AVD, AVR4(C122S) and dcAVD showed a slow dissociation. For dcAVD/AVR4, a clearly biphasic dissociation process was observed. The analysis of the first part of the measurement (0–300 s, inset) reveals an estimate for the dissociation rate constant of 1.13×10^−3^ s^−1^ for the rapid dissociation phase of dcAVD/AVR4, which is about 100× greater when compared to avidin or AVR4(C122S).

Fluorescently labeled biotin and surface plasmon resonance (SPR) biosensor were used to study the biotin-binding characteristics of dcAVD/AVR4. In the experiment with fluorescently labeled biotin a bi-phasic ligand-dissociation process was detected, where roughly 50% of the protein subunits released biotin with rapid (k_diss_ = 1.1×10^−3^ s^−1^) dissociation kinetics (Figure 6). It is probable that the rapid biotin-dissociation phase was associated with the AVR4-derived cp65 domain. As we have previously shown, the biotin-binding affinity of the circularly permuted avidin cp65 is slightly less than wt AVD. In contrast, the cp54 version appears to behave more like wt AVD in terms of biotin-binding [9]. The previous studies have shown that the AVR4 protein has a slightly lower biotin-binding affinity (K_d_ = 3.6×10^−14^ M) than avidin (K_d_ = 1.1×10^−16^ M) [25]; however, the measured increase in the biotin dissociation rate of dcAVD/AVR4 was higher than expected based on previous studies, possibly reflecting the cooperativity between different parts of the biotin-binding site [26], [27].

In the SPR analysis using the Biacore X instrument, the biotin derivative 2–iminobiotin was coupled to the sensor chip using amino-coupling chemistry. For the dcAVD/AVR4, we measured an apparent equilibrium dissociation constant of 9.6×10^−6^ M, which is greater than the constant measured for the parental proteins (wtAVD K_d_ = 1.2×10^−7^ M, AVR4(C112S) K_d_ = 7.2×10^−7^ M). The binding of dcAVD strongly resembled the wtAVD that was analyzed in the SPR assay in our preceding study [9].

The results from the fluorescent biotin interaction and the SPR analyses were consistent with each other; however, for the SPR measurements, the 2-iminobiotin ligand was attached onto the surface, and therefore, the interaction between the immobilized ligand and free protein was measured. For the measurements using the fluorescently labeled biotin, the ligand moved freely in solution. Therefore, SPR analysis could overestimate the binding affinity of chimeric dcAVDs because the protein might preferably bind the immobilized ligand with the subunit that has the greater binding affinity.

The biotin-binding properties of dcAVD/AVR4 and those measured for dcAVDs carrying point mutations in the biotin-binding site [11] suggested that the modification of the loop connecting beta strands 5 and 6 in the C-terminal domain of dual-chain avidin had negative effects on biotin-binding. In both studies, the dissociation rate constant for the C-terminal domain (cp 65, see Figure 1) was decreased more than expected, based on the sequence of the domain. Therefore, it would be logical to engineer the C-terminal domain of the dcAVD for novel characteristics while preserving the wt AVD-like characteristics of the N-terminal domain (cp54). This would exploit the existing biotinylated molecular tools maximally in dcAVD-based applications.

### Conclusions

Dual-chain avidin is an example of the complicated genetic engineering possible with the robust structure of avidin [9]. In the current study, the usability of dcAVD was improved by creating chimeric dual-chain avidin proteins. As a fusion partner with wt AVD, we used three different proteins from the avidin protein family: streptavidin, AVR2 and AVR4. The most successful was a chimeric tandem fusion of circularly permutated wt AVD and circularly permuted AVR4. Enhanced protein expression and thermal stability resulted when compared to the original dcAVD. Also, the PCR amplification was more straightforward when using chimeric dual chain fusion. Closer analyses of dcAVD/AVR4 protein showed that the molecule exhibited heterogenous biotin-binding. This might be advantageous in applications where two different biotin-binding affinities are needed. To further this technique, the dcAVD/AVR4 format can be combined with other genetically engineered avidins with modified or completely new binding properties. For instance, avidins with steroid-binding have been developed in our group by random mutagenesis and selected by phage display (Riihimäki et al., manuscript). By combining these modified avidins to a dcAVD or single-chain (scAVD, [28]) avidin platform, it will be possible to develop avidin-based receptors with alternative binding affinities or with multiple ligand specificities to be used in *in vitro* diagnostics or in nanotechnology.

## Materials and Methods

### Evaluation of the PCR performance of the chimeric dcAvd fusions

PCR amplification of the chimeric tandem fusion genes was performed to evaluate their suitability for genetic modification. The first PCR experiment was performed using four conditions; the second PCR experiment was performed using two conditions (Table S1). *Pfu* polymerase (Fermentas) was used and reactions were performed following the manufacturer's instructions. All PCR products were analyzed by 1.5% agarose gel electrophoresis using a 1-kbp DNA ladder as a standard.

### Production of chimeric dcAVD fusion proteins

DNA constructs of circularly permutated avidin cp54 were fused to circularly permuted streptavidin, AVR2 and AVR4 (cp65 SA/AVR2/AVR4) [9]. Circularly permuted genes were inserted into the pBVboostFG plasmid containing the region encoding the OmpA signal sequence, and the plasmid was transformed into chemically competent *E. coli* TOP10 cells (Invitrogen) by the standard heat-shock method. The plasmids were harvested using the Qiagen Plasmid Miniprep kit according to the manufacturer's instructions, and the inserts were verified by DNA sequencing. The amino acid sequences of the recombinant proteins and the cloning cassette are shown in Figure 1.

For protein production, the plasmids were transformed into *E. coli* BL21-AI cells (Invitrogen) by the heat-shock method. After overnight induction at 26°C, the avidin proteins were purified by affinity chromatography on a 2-iminobiotin column (Affiland, Liège, Belgium) [19], [29]. The avidin concentration was measured with a NanoDrop™ 3300 spectrometer using a molar absorption coefficient (for dcAVD/AVR4; ε = 50 880 M^−1^ cm^−1^ and M = 29 045 g/mol, for dcAVD/AVR2; ε = 48 320 M^−1^ cm^−1^ and M = 28 866 g/mol and for dcAVD/SA ε = 65 980 M^−1^ cm^−1^ and M = 28 316 g/mol) at 280 nm. The produced hybrid dcAVD proteins were analyzed by SDS-PAGE stained with Coomassie Brilliant Blue.

### Production of dcAvd/AVR4 by pilot-scale fermentation

For pilot-scale fermentation of dcAVD/AVR4 protein, the Infors Labfors 3 fermentor was used. LB medium containing gentamycin (7 µg/ml) and 0.1% glucose (w/v) was used for fermentation, supplemented with antifoam agent struktol J647 (Schill & Seilacher, Hamburg, Germany). Overnight bacterial culture in LB medium was used for inoculation. In the beginning of fermentation, the pO_2_ of dissolved oxygen was 0%, stirring speed 500 rpm and OD_600_ 0.288. Fermentation was performed at 28°C. The level of oxygen was slowly raised to obtain 20% dissolved oxygen (pO_2_). A feedback loop to maintain the oxygen level at 20% was set by adjusting the stirring speed ranging from 150 to 1100 rpm. Pumping of the feed solution (50% glycerol, 2.5 g/l MgSO_4_, 33 ml/h) was initiated at OD∼1. The induction of protein production was performed at OD∼1.5 by adding 0.25 mM IPTG (Fermentas) and 0.2% (w/v) L–arabinose (Sigma). Induction was continued for 24 h. The cells were harvested by centrifugation (5000×g, 15 min, 20°C), lysed by sonication and subjected to 2-iminobiotin affinity chromatography as previously described [19].

### Oligometric state of the produced chimeric dual chain fusions

Size-exclusion chromatography was used to determine the oligomeric state of the produced fusion proteins with an Äkta Purifier P10 instrument (Amersham Biosciences) equipped with a Tricorn™ High Performance Column Superdex™ 75 10/300 GL. A 50-µg sample of protein diluted in liquid phase (50 mM NaPO_4_ pH 7, 650 mM NaCl) was used. Bio-Rad standard proteins ranging from 1.5 to 67 kDa were used to calibrate the column.

### Thermostability of dcAVD/AVR4 protein

For SDS-PAGE thermostability analysis, the purified proteins were first *in situ* acylated [30]. Sulfosuccinimidyl acetate (Pierce #26777) was used for protein acylation. D-biotin (140 µM) was added to half the samples, and an equal volume of water was added to the other half. Samples were diluted with SDS- and 2-mercaptoethanol-containing sample buffer and incubated at different temperatures (without biotin: RT (20–22°C), 40°C, 50°C, 60°C, 70°C, 100°C; with biotin: RT, 70°C, 80°C, 90°C, 100°C) for 20 min. After incubation, the samples were analyzed by SDS-PAGE stained with Coomassie Brilliant Blue. Thermostability of AVR4(C122S) protein was also determined. The temperatures used for AVR4(C122S) protein were without biotin: RT, 60°C, 70°C, 80°C, 90°C, 100°C; and with biotin: RT, 80°C, 90°C, 100°C, 120°C.

The thermodynamics of dcAVD/AVR4 unfolding was characterized by differential scanning calorimetry (DSC) [31]. In the DSC measurements, wt dcAVD was used as a control. The proteins were analyzed in 50 mM sodium phosphate buffer (pH 7.0) containing 100 mM of sodium chloride. The temperature was increased from 20 to 130°C with a 1°C/min heating rate.

### Determination of the dissociation rate constant of fluorescent biotin

The dissociation rate constant of conjugated biotin was examined by fluorescence spectroscopy using fluorescently labeled biotin (Bf560–BTN, Arcdia, Turku, Finland). A 50-nM biotin-fluorochrome solution diluted in 50 mM sodium phosphate buffer (pH 7.0) containing 650 mM of sodium chloride was mixed with the protein solution (50 nM or 100 nM), followed by incubation (5 min). The dissociation process was illuminated by adding 100-fold excess of D-biotin (5 µM), which was measured after 1 hour. The measurements were performed at 25°C or 50°C with QuantaMaster Spectrofluorometer System equipped with thermostated water bath [19].

### Determination of 2-iminobiotin binding kinetics by surface plasmon resonance biosensor analysis

The SPR analysis was performed for wt AVD, dcAVD/AVR4 and AVR4 proteins. The CM5 chips functionalized with 2-iminobiotin were used for the interaction analysis. The Biacore X instrument (GE Healthcare) was used and the measurements were performed at 25°C using 50 mM sodium carbonate buffer (pH 11) containing 1 M of sodium chloride as the running buffer. The kinetic data were calculated from the sensograms using Langmuirian binding model implemented in the BIAevaluation® program.

### Molecular modeling and molecular dynamics simulations

The predicted models of chimeric dual-chain fusion proteins were generated by exploiting the existing 3-D structures of dcAVD (PDB 2C4I), SA (PDB 1MK5), AVR2 (PDB 1WBI) and AVR4 (PDB 1Y53) as templates. The sequence forming the cp65-subunit of dcAVD (PDB 2C4I) was first replaced with a corresponding sequence from another protein that had its sequence reorganized equally (for details, see Figure 1 and [9]). The program Modeller 9v2 [32] was then used to generate a homology model of the pseudodimer. Structural water molecules were included according to their template structures, and the following structures were also used to position the structural water molecules: PDB 1SLF, PDB 1AVE, and PDB 2AVI. The pseudotetrameric forms were then generated by positioning two pseudodimers by structural superimposition with BODIL [33]. The generated homology models were subjected to molecular dynamics simulation using the program NAMD 2.6 and the CHARMM22 force field [34]. First, the models (including structural waters extracted from PDB-files) were placed in a box filled with TIP3 waters (box size approximately 80 Å×80 Å×70 Å) using the SOLVATE algorithm in the program VMD 1.8.6 [35]. Na^+^ or Cl^−^ ions were added to neutralize the system. The complete systems contained 58377 (dcAVD/AVR4), 56803 (dcAVD/AVR2), and 53207 (dcAVD/SA) atoms. The systems were then subjected to minimization as follows: the first minimization was performed by fixing all the protein atoms and allowing water molecules to move according to minimization procedure implemented in NAMD for 4000 steps. The second 4000 step minimization was performed by releasing all the atoms in the system except Cα atoms. Finally, the system was minimized without constraints for 4000 steps.

The minimized system was thermalized by increasing the temperature to 310 K in 31 ps with a Berendsen barostat (1 atm). This step was followed by the production of the coordinate trajectories under NPT conditions using the Berendsen barostat (1 atm) and the Berendsen thermostat at 310 K. The resulting data were analyzed using the programs VMD 1.8.7, PyMOL 1.3, and MS Excel 2010. RMSF calculations were run such that the Cα atoms of one peptide chain were first aligned, and RMSF (deviation over the last 10 ps) values were calculated for the Cα atoms of the aligned chain. RMSF values were taken every 5 ps for the last 3 ns of equilibration (600 time points) and averaged. The force-field interaction energies between domains of dcAVDs were calculated from the simulation trajectories by the NAMD 2.7 program using 5-ps time step.

## Supporting Information

Figure S1 **Oligomeric state of the dcAVD/AVR4 protein determined by gel filtration.** Gel-filtration chromatography analysis showed a main peak corresponding to a dimeric (pseudotetrameric) dcAVD/AVR4 (estimated molecular weight of 46 kDa). Additionally, some higher molecular weight species are detected.
(TIF)

Figure S2 **The SDS-PAGE analysis of the pilot-scale production and the 2-iminobiotin purification of the dcAVD/AVR4 protein.** A Labfors Infors 3 bioreactor was used for a pilot-scale production of the dcAVD/AVR4 protein. The pilot-scale fed-batch fermentation in standard LB medium yielded over 5 mg of the pure dcAVD/AVR4 protein per liter of production medium. A, chicken avidin (10 µg); T, total sample from culture; L, unbound fraction after incubation with 2-iminobiotin; f2–f17 samples of elution fractions. The PageRuler™ Plus prestained protein ladder (Fermentas) was used as a molecular weight standard.
(TIF)

Table S1 **Conditions used in the PCR amplification analysis.** In the first PCR experiment, four conditions were used by varying annealing and elongation times. In the second PCR experiment, two different conditions were used as described in the table.
(DOC)
