# Supplementary figures and images for: Construction of Chimeric Dual-Chain Avidin by Tandem Fusion of the Related Avidins
Source: PLoS One. 2011 May 31;6(5):e20535. doi: 10.1371/journal.pone.0020535 (PMC3105096; doi:10.1371/journal.pone.0020535)

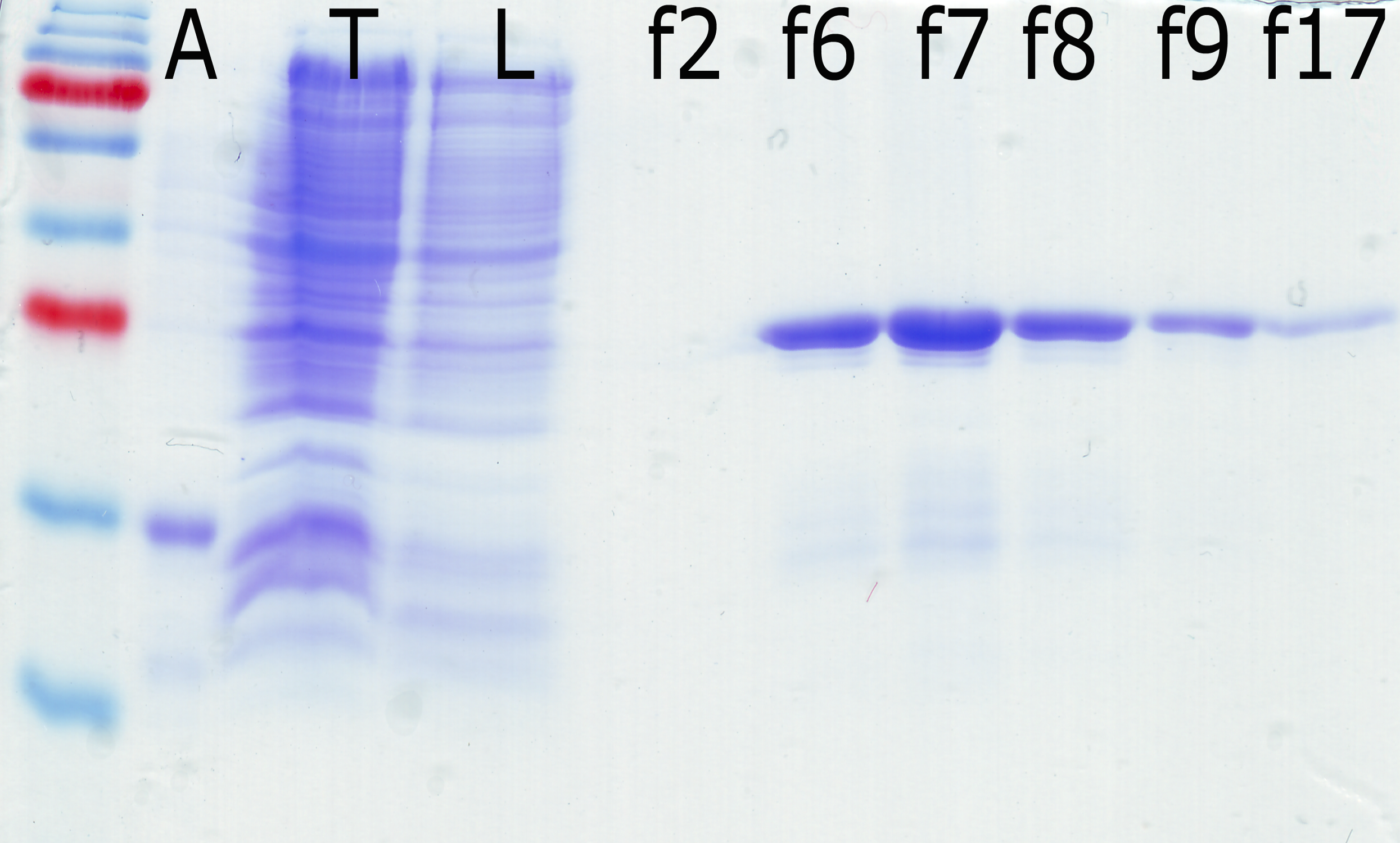

Supplement: Figure S1 — Oligomeric state of the dcAVD/AVR4 protein determined by gel filtration. Gel-filtration chromatography analysis showed a main peak corresponding to a dimeric (pseudotetrameric) dcAVD/AVR4 (estimated molecular weight of 46 kDa). Additionally, some higher molecular weight species are detected. (TIF) [file pone.0020535.s001.tif]

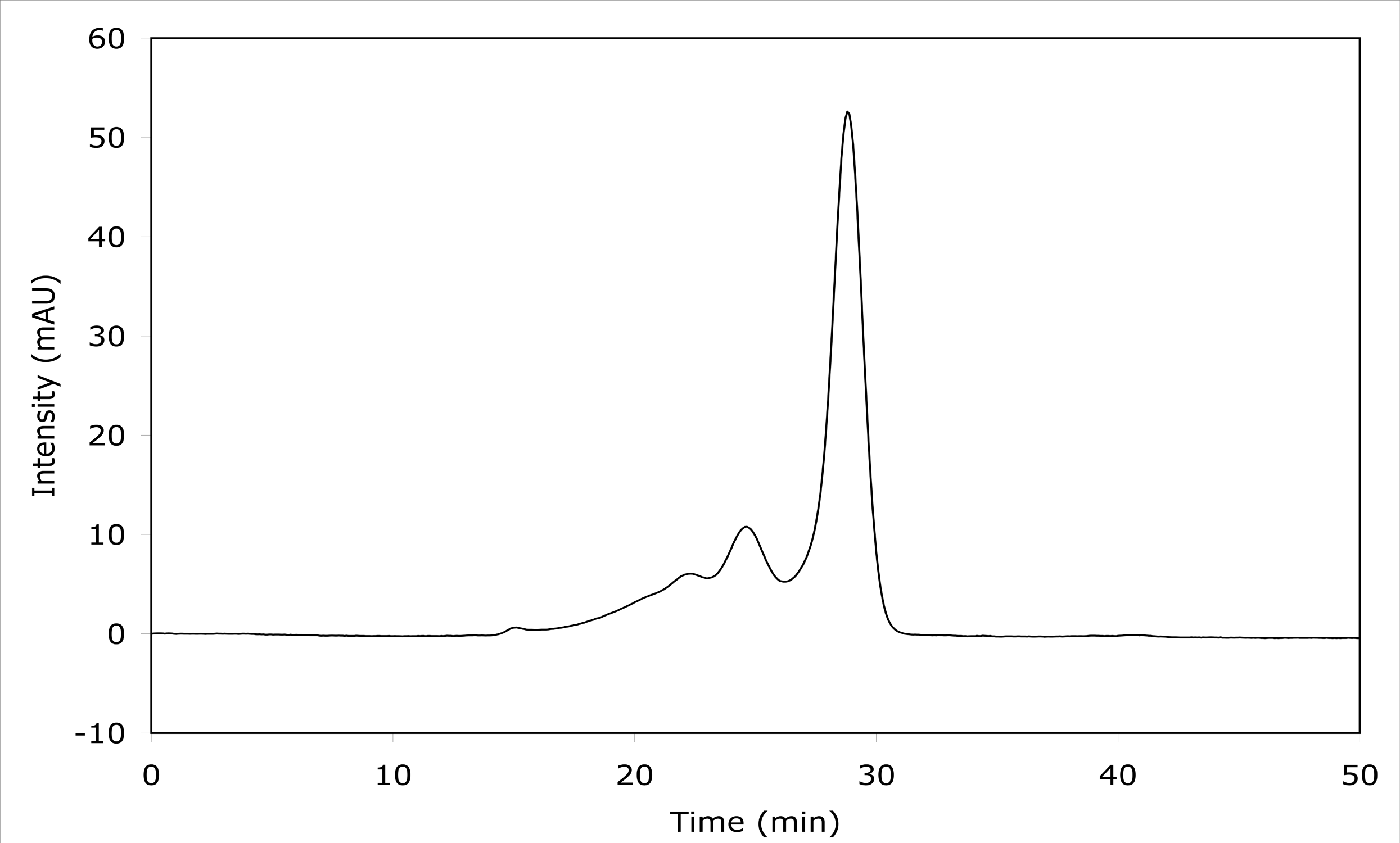

Supplement: Figure S2 — The SDS-PAGE analysis of the pilot-scale production and the 2-iminobiotin purification of the dcAVD/AVR4 protein. A Labfors Infors 3 bioreactor was used for a pilot-scale production of the dcAVD/AVR4 protein. The pilot-scale fed-batch fermentation in standard LB medium yielded over 5 mg of the pure dcAVD/AVR4 protein per liter of production medium. A, chicken avidin (10 µg); T, total sample from culture; L, unbound fraction after incubation with 2-iminobiotin; f2–f17 samples of elution fractions. The PageRuler™ Plus prestained protein ladder (Fermentas) was used as a molecular weight standard. (TIF) [file pone.0020535.s002.tif]
