# Supplementary material for: Construction of Chimeric Dual-Chain Avidin by Tandem Fusion of the Related Avidins
Source: PLoS One. 2011 May 31;6(5):e20535. doi: 10.1371/journal.pone.0020535 (PMC3105096; doi:10.1371/journal.pone.0020535)
Supplement: Table S1 — Conditions used in the PCR amplification analysis. In the first PCR experiment, four conditions were used by varying annealing and elongation times. In the second PCR experiment, two different conditions were used as described in the table. (DOC) [file pone.0020535.s003.doc]

**Table S1.** **Conditions used in the PCR amplification analysis.**

In the first PCR experiment, four conditions were used by varying annealing and elongation times. In the second PCR experiment, two different conditions were used as described in the table.

| PCR 1 | Denaturation | Annealing | Elongation | Repeated cycles |
| --- | --- | --- | --- | --- |
| I | 95˚C, 60s | 52˚C, 60s | 72˚C, 5s | ×25 |
| II | 95˚C, 60s | 52˚C, 60s | 72˚C, 15s | ×25 |
| III | 95˚C, 60s | 52˚C, 30s | 72˚C, 5s | ×25 |
| IV | 95˚C, 60s | 52˚C, 30s | 72˚C, 15s | ×25 |
| PCR 2 |  |  |  |  |
| I | 95˚C, 60s | 52˚C, 30s | 72˚C, 5s | ×25 |
| II | 95˚C, 60s | 52˚C, 30s | 72˚C, 15s | ×25 |
